# The protein phosphatase PP6 promotes RIPK1-dependent PANoptosis

**DOI:** 10.1186/s12915-024-01901-5

**Published:** 2024-05-29

**Authors:** Ratnakar R. Bynigeri, R. K. Subbarao Malireddi, Raghvendra Mall, Jon P. Connelly, Shondra M. Pruett-Miller, Thirumala-Devi Kanneganti

**Affiliations:** 1 Department of Immunology, St. Jude Children’s Research Hospital, Memphis, TN 38105 USA; 2 Current affiliation: Biotechnology Research Center, Technology Innovation Institute, Abu Dhabi, United Arab Emirates; 3 Center for Advanced Genome Engineering (CAGE), St. Jude Children’s Research Hospital, Memphis, TN 38105 USA

**Keywords:** CRISPR, PANoptosis, Caspase, TAK1, RIPK1, PP6 complex, PPP6C, PPP6R1, PPP6R2, PPP6R3

## Abstract

**Background:**

The innate immune system serves as the first line of host defense. Transforming growth factor-β–activated kinase 1 (TAK1) is a key regulator of innate immunity, cell survival, and cellular homeostasis. Because of its importance in immunity, several pathogens have evolved to carry TAK1 inhibitors. In response, hosts have evolved to sense TAK1 inhibition and induce robust lytic cell death, PANoptosis, mediated by the RIPK1-PANoptosome. PANoptosis is a unique innate immune inflammatory lytic cell death pathway initiated by an innate immune sensor and driven by caspases and RIPKs. While PANoptosis can be beneficial to clear pathogens, excess activation is linked to pathology. Therefore, understanding the molecular mechanisms regulating TAK1 inhibitor (TAK1i)-induced PANoptosis is central to our understanding of RIPK1 in health and disease.

**Results:**

In this study, by analyzing results from a cell death-based CRISPR screen, we identified protein phosphatase 6 (PP6) holoenzyme components as regulators of TAK1i-induced PANoptosis. Loss of the PP6 enzymatic component, PPP6C, significantly reduced TAK1i-induced PANoptosis. Additionally, the PP6 regulatory subunits PPP6R1, PPP6R2, and PPP6R3 had redundant roles in regulating TAK1i-induced PANoptosis, and their combined depletion was required to block TAK1i-induced cell death. Mechanistically, PPP6C and its regulatory subunits promoted the pro-death S166 auto-phosphorylation of RIPK1 and led to a reduction in the pro-survival S321 phosphorylation.

**Conclusions:**

Overall, our findings demonstrate a key requirement for the phosphatase PP6 complex in the activation of TAK1i-induced, RIPK1-dependent PANoptosis, suggesting this complex could be therapeutically targeted in inflammatory conditions.

**Supplementary Information:**

The online version contains supplementary material available at 10.1186/s12915-024-01901-5.

## Background

The innate immune system plays a fundamental role in maintaining tissue homeostasis and establishing the first line of defense against pathogens and sterile inflammatory agents [1]. Pathogen-associated molecular patterns (PAMPs), damage-associated molecular patterns (DAMPs), and homeostasis-altering molecular processes (HAMPs) are sensed by genetically encoded pattern recognition receptors (PRRs) to initiate inflammatory responses and cell death [2, 3]. Downstream of PRR engagement, transforming growth factor-β (TGF-β)–activated kinase 1 (TAK1) is a key signaling node to drive the expression of inflammatory molecules and cell death, which are critical for host defense and cellular homeostasis [4–7]. Given these critical functions, many pathogens carry TAK1 inhibitors to evade or disable host defenses. A classic example comes from *Yersinia*, which uses its type III secretion system to release a highly virulent *Yersinia* outer protein J (YopJ) that inhibits TAK1 in the host innate immune cells. To counteract pathogens, hosts sense TAK1 inhibition to induce robust lytic cell death, PANoptosis, mediated by the RIPK1-PANoptosome, which contains the NLRP3 inflammasome, caspase-8, FADD, and RIPK3 and drives cell death [8–10].

PANoptosis is a unique innate immune inflammatory lytic cell death pathway initiated by an innate immune sensor and driven by caspases and RIPKs [8, 11]. While the activation of PANoptosis in response to TAK1 inhibition promotes host defense, excess activation drives inflammation and pathogenesis. Therefore, the activation of TAK1 inhibitor (TAK1i)-induced PANoptosis involves a complex network of regulatory mechanisms. While it is known that regulation occurs through the TNFR1-RIPK1 signaling axis and involves the Rag-Regulator complex [5, 6, 8–10], our understanding of the molecular mechanisms regulating TAK1i-mediated PANoptosis remains incomplete.

To advance the mechanistic understanding of TAK1i-induced PANoptosis, we evaluated the novel regulators previously identified in a whole genome cell death-based CRISPR knockout screen in immortalized bone marrow-derived macrophages (iBMDMs) using TAK1i as the trigger [12]. In this study, we identified the core components of the protein phosphatase 6 (PP6) holoenzyme PPP6C and PPP6R3 (also known as PP6C and PP6R3) as highly enriched CRISPR screen hits. Loss of PPP6C significantly reduced TAK1i-induced cell death and the activation of PANoptosis molecules. In contrast, combined deletion of all three PP6 regulatory subunits, PPP6R1, PPP6R2, and PPP6R3, was needed to inhibit TAK1i-induced cell death, suggesting that these molecules act redundantly in the TAK1 regulatory pathway. Additionally, we observed that the phosphatase PP6 led to a reduction in the inhibitory S321 phosphorylation of RIPK1, tipping the balance toward the pro-death S166 auto-phosphorylation of RIPK1 and triggering RIPK1-dependent PANoptosis in response to TAK1 inhibition. Overall, our findings identify a critical new regulator in the TAK1 inhibition-mediated inflammatory cell death pathway, suggesting PP6 may be a potential therapeutic target to reduce inflammation and certain forms of immunopathology.

## Results

### A whole genome CRISPR screen identified the phosphatase PP6 as a critical regulator of TAK1 inhibition-induced cell death

**Fig. 1:**
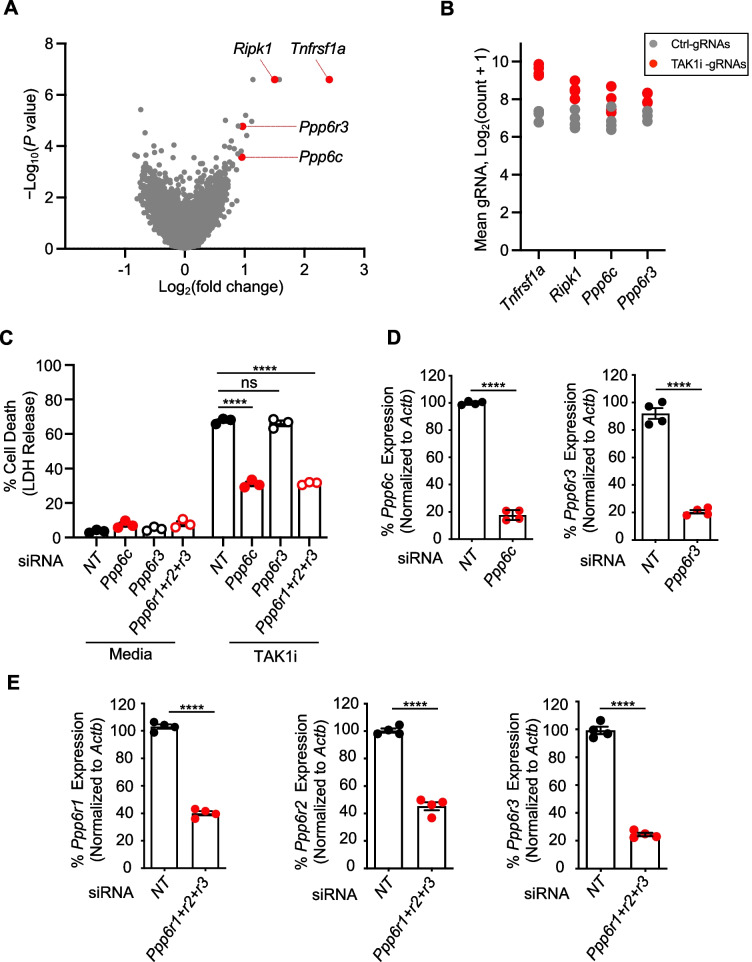
Whole genome CRISPR screen identifies PPP6C and PPP6R3 as key regulators of TAK1 inhibition-induced cell death. **A** Volcano plot presenting the log_2_ mean fold change for *Ripk1*, *Tnfrsf1a*,* Ppp6r3*, and *Ppp6c* gRNAs, along with all other tested gRNAs, in the TAK1 inhibitor (TAK1i) CRISPR screen, following the treatment of immortalized bone marrow-derived macrophages (iBMDMs) carrying gRNAs with TAK1i for 24 h. **B** Scatter plot showing the enrichment of all four gRNAs targeting *Ripk1*, *Tnfrsf1a*,* Ppp6r3*, and *Ppp6c* in the pool of iBMDMs carrying gRNAs from the whole genome CRISPR screen. **C** Percent cell death as measured by lactate dehydrogenase (LDH) release in primary BMDMs treated with siRNA against *Ppp6c*,* Ppp6r3*, or the combination of *Ppp6r1*, *Ppp6r2*, and *Ppp6r3* in response to TAK1i treatment. **D** Relative expression of *Ppp6c* and *Ppp6r3* in BMDMs treated with siRNA against *Ppp6c and Ppp6r3*, respectively*.* **E** Relative expression of *Ppp6r1*, *Ppp6r2*, and *Ppp6r3* in the BMDMs treated with siRNAs to silence the three PP6 regulatory subunits together*.* The knockdown efficiency is expressed as a percentage of the respective gene expression normalized to *Actb* expression. The data presented are representative of three or more independent experiments (**C**–**E**). The data are shown as mean ± SEM (**C**–**E**). Statistical analysis was performed using Student’s *t*-test and two-way ANOVA. ns, not significant; ****P < 0.0001. NT: non-targeting siRNA; *Ppp6r1+r2+r3*: BMDMs treated with siRNAs against *Ppp6r1*, *Ppp6r2*, and* Ppp6r3*

Given the established role of TAK1 as a central hub for cellular homeostasis and cell death [4–9], understanding the complex regulation of TAK1i-mediated PANoptosis is central to health and disease. A previous study to identify regulators of TAK1i-induced cell death used a whole genome CRISPR screen in iBMDMs and identified several gRNAs that were enriched in cells that survived following 24 h of TAK1i treatment [12]. In this cell death screen, the genes corresponding to the enriched gRNAs represent the positive regulators required for the induction of TAK1i-induced cell death. To understand more about these novel regulators of TAK1i-induced cell death, we evaluated the hits from the previously generated CRISPR screen data and found that TNFR1 (TNFRSF1A) and RIPK1, two well-known regulators of TAK1i-mediated cell death, were the top hits from the screen (Fig. 1A). Furthermore, we also identified the enrichment of two of the phosphatase PP6 holoenzyme components, *Ppp6c* and *Ppp6r3* (Fig. 1A). Additional analyses of the individual gRNA counts demonstrated that all four gRNAs corresponding to the genes *Ppp6c* and *Ppp6r3* were detected in the TAK1i-treated cell pools, with the majority of them showing increased mean total gRNA counts compared to the control samples (Fig. 1B). These results suggest that deletion of *Ppp6c* or *Ppp6r3* with any of their gRNAs was sufficient to protect the cells from TAK1i-induced cell death. Together, the CRISPR MageCK analysis suggests that *Ppp6c* and *Ppp6r3* were key molecules involved in driving TAK1i-induced cell death.

### The phosphatase PPP6C and its regulatory subunits are essential for TAK1i-induced cell death

The phosphatase PP6 belongs to the PPP superfamily of serine/threonine phosphatases [13, 14]. PPP6C is the catalytic subunit, and it interacts with its regulatory subunits, the PPP6Rs (PPP6R1, PPP6R2, and PPP6R3), to form the fully functional PP6 protein phosphatase holoenzyme complex. PP6 plays key roles in cell cycle progression [15, 16], and it has been implicated in restricting innate immune hyperactivation. Specifically, PP6 inhibits (i) TAK1 activation by dephosphorylating T187 in the TAK1 activation loop [17], (ii) the cGAS-STING pathway by dephosphorylating cGAS and STING at S366 [18, 19], and (iii) NF-κB signaling [14]. However, PP6-mediated dephosphorylation also promotes the activation of RIG-I and can therefore positively regulate protective innate immune signaling and host defense against viral infections [20]. PP6 has also been linked to the modulation of LUBAC-mediated M1-ubiquitination of RIPK1 to enable RIPK1 activation and the induction of cell death [21, 22]. Based on these connections between PP6 and innate immune activation, combined with our finding that PP6 components are potential regulators of TAK1i-induced cell death from the CRISPR screen (Fig. 1A–B), we sought to validate the function of these components using primary murine macrophages. We transfected primary bone marrow-derived macrophages (BMDMs) with control (NT, non-targeting) or gene-specific siRNAs to knockdown the expression of *Ppp6c* and *Ppp6r3*. Consistent with the findings from the TAK1i CRISPR screen in iBMDMs (Fig. 1A–B), the primary BMDMs transfected with NT siRNA showed robust cell death in response to TAK1i treatment, while the BMDMs transfected with siRNA targeting *Ppp6c* exhibited a significant reduction in cell death (Fig. 1C), as well as reductions in *Ppp6c* transcript and PPP6C protein expression (Fig. 1D, Additional File 1: Fig. S1A, Additional File 2: Uncropped blots). However, the siRNA-based knockdown of *Ppp6r3* failed to protect the cells from TAK1i-induced cell death (Fig. 1C), despite a reduction in *Ppp6r3* expression and PPP6R3 protein level in the siRNA-treated BMDMs (Fig. 1D, Additional File 1: Fig. S1B, Additional File 2: Uncropped blots). Previous studies have demonstrated that the phosphatase PPP6C can interact with other regulatory subunits [23], potentially bypassing the need for PPP6R3 in primary BMDMs. Therefore, we next assessed the potential roles of other regulatory subunits. However, similar to our results with PPP6R3, siRNA-mediated silencing of the other PP6 regulatory components, *Ppp6r1* and *Ppp6r2*, had no effect on cell death, despite a reduction in their transcript and protein expression levels (Additional File 1: Fig. S1B–C, Additional File 2: Uncropped blots). To determine whether there was functional redundancy between the regulatory subunits that allowed the overall regulatory functions to be maintained despite the loss of an individual subunit, we then performed siRNA-mediated knockdown of the three regulatory subunits of phosphatase PP6 together. We found that the combined knockdown of *Ppp6r1*, *Ppp6r2*, and *Ppp6r3* resulted in a significant reduction in each component’s expression (Fig. 1E) and reduced TAK1i-induced cell death, comparable to the reduction observed in *Ppp6c* siRNA-treated cells (Fig. 1C). Together, these data suggest that the catalytic subunit PPP6C was required for TAK1i-mediated cell death, while its regulatory subunits PPP6R1, PPP6R2, and PPP6R3 acted redundantly to form a fully functional PP6 phosphatase enzyme that drives TAK1i-mediated cell death.

### Loss of the phosphatase PPP6C or its regulatory subunits abrogates the induction of TAK1i-induced PANoptosis

**Fig. 2:**
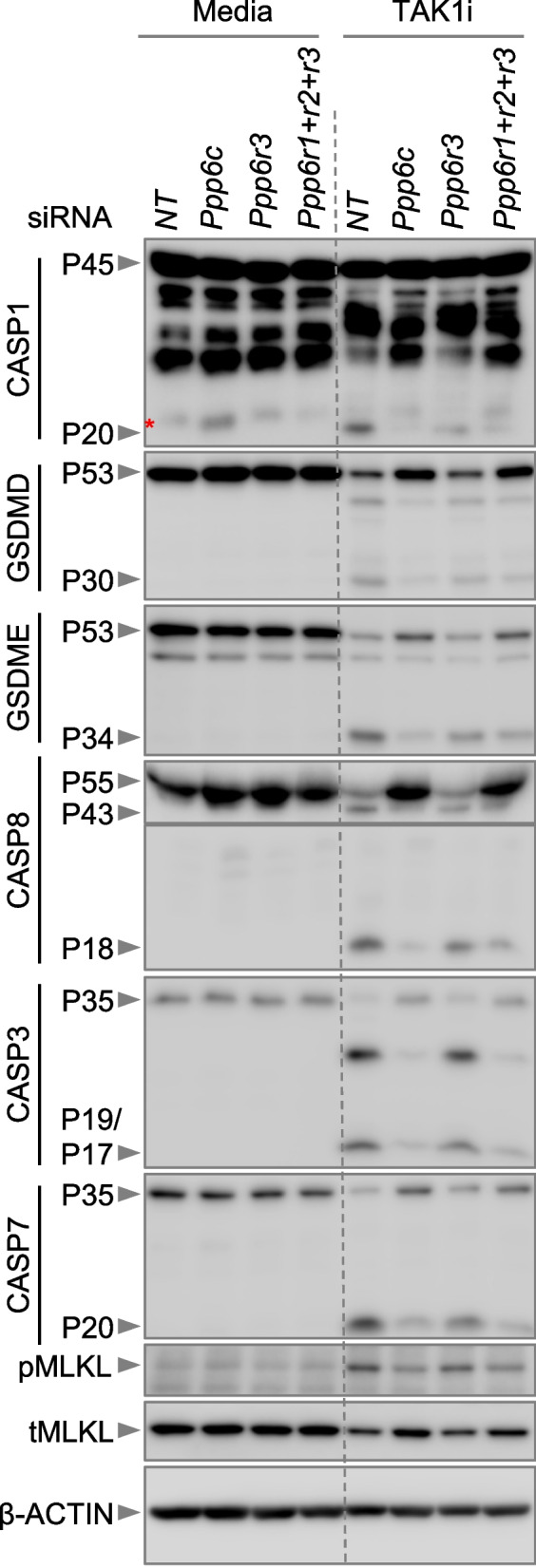
The PP6 complex subunits are required for TAK1 inhibition-induced PANoptosis. Immunoblots for pro- (P45) and cleaved caspase-1 (P20; CASP1), pro- (P53) and activated (P30) gasdermin D (GSDMD), pro- (P53) and activated (P34) gasdermin E (GSDME), pro- (P55) and cleaved caspase-8 (P43/18; CASP8), pro- (P35) and cleaved caspase-3 (P19/17; CASP3), pro- (P35) and cleaved caspase-7 (P20; CASP7), and phospho- (pMLKL) and total MLKL (tMLKL) using cell lysates from bone marrow-derived macrophages (BMDMs) treated with TAK1 inhibitor (TAK1i) for 8 h. An asterisk indicates a non-specific band near the CASP1 P20 fragment. Blots were re-probed for β-ACTIN to serve as the internal loading control. The data are representative of three independent experiments. NT: non-targeting siRNA; *Ppp6r1+r2+r3*: BMDMs treated with siRNAs against *Ppp6r1*, *Ppp6r2*, and* Ppp6r3*

As the CRISPR screen and validation analyses identified a key role for PP6 components in TAK1i-induced cell death, we next sought to examine the effect of the PP6 subunits on the biochemical activation of PANoptosis molecules. Similar to the previous findings [5, 6, 12], we observed that TAK1i treatment induced activation of cell death molecules including caspase-1, gasdermin (GSDM) D and E, caspase-8, caspase-3, caspase-7, and MLKL in the NT siRNA-transfected BMDMs (Fig. 2, Additional File 2: Uncropped blots). The siRNA-mediated knockdown of *Ppp6c*, as well as the collective knockdown of *Ppp6r1*,* Ppp6r2*, and *Ppp6r3*, reduced the activation of each of these molecules (Fig. 2, Additional File 2: Uncropped blots). The siRNA-mediated knockdown of *Ppp6r3* resulted in a slight reduction in the activation of caspase-1 and GSDMD/GSDME; however, we consistently observed that the activation of caspase-8, caspase-3, caspase-7, and MLKL was comparable or only marginally reduced in the *Ppp6r3 *siRNA-treated cells compared with the NT control (Fig. 2, Additional File 2: Uncropped blots). Similarly, siRNA-mediated knockdown of *Ppp6r1* and *Ppp6r2* decreased the activation of caspase-1 and caspase-7 and slightly decreased the activation of MLKL, with no substantial changes in the activation of other cell death proteins (Additional File 1: Fig. S1D, Additional File 2: Uncropped blots). These observations, together with the cell death data (Fig. 1C, Additional File 1: Fig. S1C), demonstrate that the PP6 regulatory subunits have complementary roles and functional redundancy to promote activation of RIPK1-dependent PANoptosis in TAK1i-treated cells.

### The PP6 complex negatively regulates inhibitory phosphorylation of RIPK1 to drive PANoptosis

**Fig. 3:**
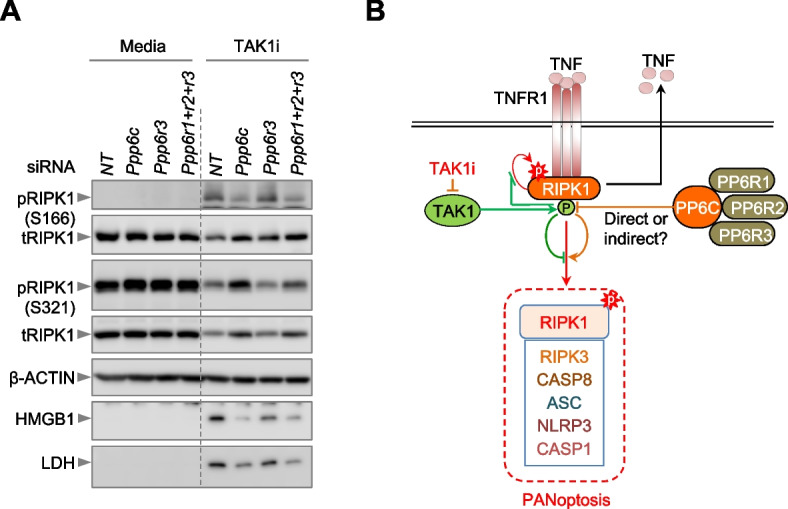
The PP6 complex negatively regulates inhibitory phosphorylation of RIPK1 to drive PANoptosis. **A** Immunoblots for S166 and S321 phospho-RIPK1 (pRIPK1) and total RIPK1 (tRIPK1) using cell lysates and immunoblots for high mobility group box 1 (HMGB1) and lactate dehydrogenase (LDH) using culture supernatants from bone marrow-derived macrophages (BMDMs) treated with TAK1 inhibitor (TAK1i) for 8 h. Blots were re-probed for β-ACTIN to serve as the internal loading control. The blots presented are representative of three independent experiments. NT: non-targeting siRNA; *Ppp6r1+r2+r3*: BMDMs treated with siRNAs against *Ppp6r1*, *Ppp6r2*, and *Ppp6r3*. **B** Schematic illustrating the potential mechanism for the PP6 complex to drive TAK1i-induced, RIPK1-dependent PANoptosis in BMDMs. The phosphorylation (P) marks denote the RIPK1 S166 (red, spiked) and S321 (green, rounded) phosphorylations

After confirming the role of PP6 components in the phenotypic and biochemical activation of PANoptosis, we next sought to determine how PP6 regulates TAK1i-induced cell death. As PP6 is a phosphatase, and RIPK1 phosphorylation is a key regulatory step in the TAK1 inhibition-mediated cell death pathway [5, 8], we first assessed whether PP6 components could modulate RIPK1 phosphorylation status to regulate PANoptosis. We observed that siRNA-mediated knockdown of *Ppp6c* or simultaneous knockdown of all three regulatory subunits, *Ppp6r1*,* Ppp6r2*, and *Ppp6r3*, resulted in a reduction in the cell death-activating S166 auto-phosphorylation of RIPK1 (Fig. 3A, Additional File 2: Uncropped blots). Furthermore, knockdown of the PP6 catalytic subunit *Ppp6c* or combined knockdown of all three of its regulatory subunits resulted in an increase in the cell death-inhibiting S321 phosphorylation of RIPK1 (Fig. 3A, Additional File 2: Uncropped blots). However, knockdown of *Ppp6r3* alone was insufficient to prevent S166 phosphorylation or enhance S321 phosphorylation of RIPK1 (Fig. 3A, Additional File 2: Uncropped blots), demonstrating the complementary roles of the phosphatase PP6 regulatory subunits. Moreover, consistent with the observed role for the PP6 phosphatase components in driving TAK1i-mediated lytic PANoptosis, we observed a decrease in the release of DAMPs, including lactate dehydrogenase (LDH) and high mobility group box 1 (HMGB1), in response to TAK1i treatment in the *Ppp6c* and *Ppp6r1*,* Ppp6r2*, and *Ppp6r3* siRNA-treated groups compared to the NT group (Fig. 3A, Additional File 2: Uncropped blots). Together, these results highlight the significance of the distinct phosphorylations of RIPK1 and the effect of the phosphatase PP6 on their respective levels in determining cell fate (Fig. 3B), thereby suggesting that the therapeutic modulation of the PP6 complex may be used to treat RIPK1-dependent inflammatory cell death and inflammation in infectious and inflammatory diseases.

## Discussion

TAK1 is widely recognized for its role in innate immune responses to regulate PANoptosis in a RIPK1-dependent manner [5, 8, 9, 24]. The RIPK1 kinase has emerged as a key regulator of inflammation and cell death downstream of a wide range of immune receptors [5, 6, 9, 24–28], and its optimal activation is essential to promote protective anti-microbial immune responses [9, 24, 28–30]. However, both hypo- and hyper-activation of RIPK1 can result in severe inflammatory disease [31–37]. Phosphorylation plays a critical role in fine-tuning RIPK1 activation, and the auto-phosphorylation at S166 serves as a marker for its pro-cell death function [38], whereas the phosphorylation of S25 and S321 (S320 in humans) promotes scaffold-based inflammation and survival signaling through p38^MAPK^/MK2 [30, 39, 40]. In this context, MAPKs such as TAK1, TBK1, and IKK have been shown to drive the pro-survival phosphorylation of RIPK1 [39], and their inactivation often results in severe inflammatory conditions arising from hyperactivation of RIPK1 [6, 29, 41–43]. Despite this understanding of kinase-mediated phosphorylation and regulation of RIPK1, the precise mechanisms that dephosphorylate the inhibitory S321 residue of RIPK1 to allow the activation of cell death function remain understudied.

Our study provides evidence that phosphatase PP6 and its regulatory subunits reduced RIPK1 survival phosphorylation at S321 and promoted RIPK1-dependent PANoptosis. PP6 may also regulate RIPK1’s functions by controlling its overall expression, but this remains unclear. Furthermore, siRNA-mediated knockdown of *Ppp6c* or the regulatory subunits did not fully block the TAK1i-induced cell death, suggesting that other phosphatase combinations may play a potential role, and future research is warranted to identify additional regulators of this inflammatory signaling and cell death mechanism. Moreover, a recent study demonstrated that the PPP1R3G/PP1γ phosphatase holoenzyme is essential to dephosphorylate the S25 residue of RIPK1 to drive the subsequent induction of RIPK1-dependent cell death [44]. Future work should determine whether PP6 and its subunits cooperate with PP1γ to regulate TAK1i-induced PANoptosis and whether the effect of PP6 on RIPK1 phosphorylation is direct or indirect. Additionally, a recent study has reported that the excessive production of mitochondrial ROS (mtROS) through reverse electron transport (RET) drives PANoptosis [45]. Pre-treatment with anti-RET reagents such as 1-methoxy PMS or dimethyl fumarate blocks RET-mediated mitochondrial ROS production and subsequent induction of PANoptotic cell death in BMDMs [45]. Further studies will be needed to determine the role of mtROS in TAK1i-induced cell death.

Our findings emphasize the necessity of additional investigations to identify novel phosphatases capable of negatively regulating inhibitory phosphorylation sites on RIPK1, which can be better targeted for therapeutic modulation of RIPK1 signaling. Beyond RIPK1 and its roles in inflammatory conditions, our findings are also critical in the context of *Yersinia-*induced inhibition of TAK1 function, which also drives RIPK1-dependent inflammatory cell death that may depend on PP6. The identification of the central role of PP6 in mediating TAK1i-induced PANoptosis suggests that targeting PP6 or its subunits may aid in reducing RIPK1-dependent inflammatory cell death, which is critical in sterile and infectious pathologies.

## Conclusions

Overall, our findings suggest an important role for PP6 phosphatase in TAK1i-mediated, RIPK1-driven PANoptosis. Our findings show that the PP6 complex is critical to regulating TAK1i-induced cell death, which may be clinically important in the context of targeting infections and inflammatory conditions.

## Methods

### Mice

The C57/BL6J control mice (wild type; WT) and CRISPR-Cas9 knockin mice (Rosa26-Cas9 knockin, JAX stock #024858) [46] used in the present study were maintained under specific pathogen-free conditions and bred at St. Jude Children’s Research Hospital’s Animal Resources Center. Mice were fed standard chow and maintained under a 12 h light-dark cycle. Animal studies were conducted using the standard protocols approved by the St. Jude Children’s Research Hospital Committee on the Use and Care of Animals (protocol 482).

### Whole genome iBMDM-Brie cellular library

The data generated previously from the TAK1i CRISPR screen Brie-iBMDMs were used for the analyses, following the same procedures described previously (BioProject: PRJNA973658) [12]. The calc_auc_v1.1.py (https://github.com/mhegde/) and count_spacers.py [47] were used to validate and check the presence of gRNA, followed by analysis of CRISPR KO screens using MAGeCKd-VISPR (version 0.5.7) [48].

### Isolation and stimulation of primary macrophages

To differentiate the murine bone marrow cells into macrophages, the bone marrow cells were cultured in macrophage differentiation medium (BMDM medium). BMDM medium was prepared using IMDM (12440053, Thermo Fisher Scientific), supplemented with 30% L929 cell-conditioned medium, 10% heat-inactivated fetal bovine serum (HI-FBS) (S1620, Biowest), 1% nonessential amino acids (11140-050, Thermo Fisher Scientific), and 1% penicillin-streptomycin (15070-063, Thermo Fisher Scientific). Freshly isolated murine bone marrow cells were mixed to form a single cell suspension in BMDM medium and seeded into three 15 cm^2^ non-tissue culture-treated plates, each containing 20 mL of the medium. The cells in these plates were fed with an additional 5 mL of fresh BMDM medium on days 3 and 5 to support growth and differentiation. For siRNA knockdown experiments, day 6 cells were scraped, nucleofected with the respective siRNAs, and seeded at a density of 10^6^ cells per well in 12-well culture plates in DMEM (11995-065, Gibco) containing 10% HI-FBS, 1% nonessential amino acids, and 1% penicillin-streptomycin for 2 h, followed by supplementing with an equal volume (1 mL) of fresh BMDM medium. The cells were then supplemented with fresh BMDM medium 24 h post-transfection and cultured for a total of 36–48 h from the time of transfection, followed by experimental stimulations. The TAK1 inhibitor (TAK1i, (5Z)-7-oxozeaenol, 100 nM final concentration; 17459, Cayman Chemical) was used to treat the cells for the inhibition of TAK1 kinase activity, as previously described [5, 6, 12], and the cell lysates were collected at the different time points as stated in the figure legends for the western blot analyses.

### Cell death analysis

Cell death was measured and presented as a percentage of LDH release. The LDH release was quantified using the Cyto Tox 96® Non-Radioactive Cytotoxicity assay kit (G1782, Promega), following the manufacturer’s instructions.

### Transfection

The fully differentiated WT BMDMs were transfected with the respective siRNAs using the nucleofector machine (Neon™ Transfection System, 100 μL/reaction kit, MPK10025, Thermo Fisher Scientific). Using the nucleofector settings of 1500 V and a single pulse at 20 mS, 50 μM of siGENOME SMARTpool siRNA were delivered to 10^6^ BMDMs. Similar parameters were followed for the combined knockdown of *Ppp6r1+r2+r3*, where 50 μM of each of the siGENOME SMARTpool siRNAs for the respective genes was transfected together into the BMDMs. The transfected cells were transferred to 1 mL of warm, complete DMEM and seeded in a well of the 12- and/or 24-well tissue culture plate to conduct the experimental stimulations. Subsequently, to promote growth and recovery, the transfected cells were supplemented with an equal volume of warm BMDM medium 2 h post-transfection. The spent medium was replaced with fresh BMDM at 24 h after transfection, and the cells were allowed to rest for another 12-24 h. In total, the cells were allowed to rest for 36-48 h after transfection before proceeding to experimental stimulations. The siGENOME SMARTpool siRNAs specific for mouse *Ppp6c* (M-062583-01-0010, Horizon Discovery), *Ppp6r1* (M-059588-00-0010, Horizon Discovery),* Ppp6r2* (M-044631-02-0010, Horizon Discovery), and *Ppp6r3* (M-040027-00-0010, Horizon Discovery), along with a non-targeting (NT) control siGENOME SMARTpool siRNA (D-001206-14-20, Horizon Discovery), were used in this study.

### Western blotting

The experimental cell lysates were resolved on SDS-PAGE, and western blot analyses were performed as previously reported [12]. To immunoblot the caspases studied here, the samples were prepared by mixing the cell lysates along with the culture supernatants in the lysis buffer (lysis buffer: 5% NP-40 solution in water supplemented with 10 mM DTT and protease inhibitor solution at 1× final concentration). All other proteins detected using the immunoblot analyses for intracellular components were collected by lysing the cells without the cell supernatants in the RIPA buffer. The caspase and RIPA cell lysate samples were mixed and denatured in loading buffer containing SDS and 100 mM DTT and boiled for 12 min. In addition, HMGB1 and LDH were detected in the cell culture supernatants. The culture supernatants were centrifuged at 8000 rpm for 5 min to remove cell debris and mixed with 4× loading dye at a ratio of 3:1. Then, the samples were boiled at 100 °C for 12 min and stored at − 20 °C until further use. A total of 20 μL was loaded for each of the samples in the SDS-PAGE. After resolving the proteins on SDS-PAGE, the proteins in the gel were transferred to PVDF membranes (IPVH00010, Millipore) using the Trans-Blot® Turbo™ system. Subsequently, the membranes were probed with primary antibodies against caspase-1 (AG-20B-0042; Adipogen, 1:1000), caspase-3 (#9662, Cell Signaling Technology [CST], 1:1000), cleaved caspase-3 (#9661, CST, 1:1000), caspase-7 (#9492, CST, 1:1000), cleaved caspase-7 (#9491, CST, 1:1000), caspase-8 (#4927, CST, 1:1000), GSDMD (ab209845, Abcam, 1:1000), GSDME (ab19859, Abcam, 1:1000), pMLKL (#37333, CST, 1:1000), tMLKL (AP14242B, Abgent, 1:1000), tRIPK1 (#610458, BD Biosciences, 1:1000), Ser166 pRIPK1 (#31122, CST, 1:1000), Ser321 pRIPK1 (#38662S, CST, 1:1000), PPP6C (#15852-1-AP, Proteintech, 1:1000), PPP6R1 (#PA5-44275, Invitrogen, 1:500), PPP6R2 (#TA890148, Invitrogen, 1:500), PPP6R3 (#16944-1-AP, Proteintech, 1:1000), LDH (#19987-1-AP, Proteintech, 1:1000), HMGB1 (#ab18256, Abcam, 1:1000), and β-Actin (sc-47778 HRP, Santa Cruz, 1:5000). Respective horseradish peroxidase (HRP)–conjugated secondary antibodies (anti-Armenian hamster [127-035-099], anti-mouse [315-035-047], and anti-rabbit [111-035-047], Jackson ImmunoResearch Laboratories) were used as described previously [49]. Blot images were acquired on an Amersham Imager using Immobilon® Forte Western HRP Substrate (WBLUF0500, Millipore). In certain cases, the immunoblots were stripped using stripping buffer (Restore^TM^ Western Blot Stripping buffer, 21059, Thermo Fisher Scientific) and reprobed for β-ACTIN.

### RNA isolation and qPCR analyses

Total RNA was isolated using TRIzol® reagent (#15596018, Ambion), and the concentration was measured using the NanoDrop (Thermo Fisher Scientific). A total of 500 ng of total RNA was reverse transcribed to cDNA in a 20 μL volume using a high-capacity cDNA synthesis kit (#4368813, Applied Biosystems). The cDNA was further diluted to a final volume of 200 μL using nuclease free water. The levels of *Ppp6c*, *Ppp6r1*,* Ppp6r2*, and* Ppp6r3* in the cDNA samples were quantified using qPCR analyses based on the Sybr® Green chemistry (#4367659, Applied Biosystems) in the Quant Studio™ 7 Flex Real-Time PCR machine (Applied Biosystems). The target gene expression levels were normalized to *Actb*, and the difference in the levels is represented in fold change in arbitrary units. Forward and reverse primer sequences used in the study are as follows: *Actb*, Forward: 5-CAGCTTCTTTGCAGCTCCTT-3, Reverse: 5-CACGATGGAGGGGAATACAG-3; *Ppp6c*, Forward: 5-CCGCTGGATCTGGACAAGTAT-3, Reverse: 5-ACACTGGCTGAACATTCGACT-3; *Ppp6r1*, Forward: 5-TGTGCCCAACACCTTACTGG-3, Reverse: 5-AGGAGATGTTTCACGATAGGGT-3; *Ppp6r2*, Forward: 5-CCCACGTTGACAAGCTCCT-3, Reverse: 5-GCCTTACACTCCTGCAAGATG-3; *Ppp6r3*, Forward: 5-TTGCAGACCAAGACGACATTG-3, Reverse: 5- TTCTTCACTGTCCGTACTGCC-3.

### Statistical analysis

Statistical significance was determined by *t* tests (two-tailed) for two groups and a two-way ANOVA for more than two groups. The data are represented as the mean ± SEM. The GraphPad Prism version 8.0 and 9.0 software packages were used for data analyses.

### Supplementary Information

**Additional file 1:** **Figure S1**. Individual PP6 complex regulatory subunits do not impact TAK1 inhibition-induced PANoptosis. (A–B) Immunoblots for A) PPP6C and B) PPP6R1, PPP6R2, and PPP6R3 using cell lysates from bone marrow-derived macrophages (BMDMs) following the indicated siRNA knockdowns. Blots were re-probed for β-ACTIN to serve as the internal control. C) Percent cell death as measured by lactate dehydrogenase (LDH) release in BMDMs treated with siRNA against *Ppp6r1 *or *Ppp6r2* in response to TAK1 inhibitor (TAK1i) treatment for 8 h. Statistical analysis was performed using the two-way ANOVA. ns, not significant. D) Immunoblots for pro- (P45) and cleaved caspase-1 (P20; CASP1); pro- (P53) and activated (P30) gasdermin D (GSDMD); pro- (P53) and activated (P34) gasdermin E (GSDME); pro- (P55) and cleaved caspase-8 (P43/18; CASP8); pro- (P35) and cleaved caspase-3 (P19/17; CASP3); pro- (P35) and cleaved caspase-7 (P20; CASP7); and phospho-MLKL (pMLKL) and total MLKL (tMLKL) using cell lysates from BMDMs treated with TAK1i for 8 h. An asterisk indicates a non-specific band near the CASP1 P20 fragment. Blots were re-probed for β-ACTIN to serve as the internal loading control. The data are representative of three independent experiments. NT: non-targeting siRNA.

**Additional file 2:** Uncropped blots. The uncropped, raw blots corresponding with Figs. 2, 3A, S1A, S1B, and S1D are shown with molecular weight indicators.

## Data Availability

All data generated or analyzed during this study are included in this published article or available at BioProject: PRJNA973658 (https://www.ncbi.nlm.nih.gov/bioproject/?term=PRJNA973658) [12].

## Abbreviations

ANOVA: Analysis of variance
ASC: Apoptosis-associated speck-like protein containing a caspase activation and recruitment domain
BMDM: Bone marrow-derived macrophage
Cas9: CRISPR-associated protein 9
CASP1: Caspase-1
CASP3: Caspase-3
CASP7: Caspase-7
CASP8: Caspase-8
cDNA: Complementary DNA
cGAS: Cyclic GMP-AMP synthase
CRISPR: Clustered regularly interspaced short palindromic repeats
DAMP: Damage-associated molecular pattern
DMEM: Dulbecco’s Modified Eagle Medium
DTT: Dithiothreitol
FADD: Fas-associated via death domain
gRNA: Guide RNA
GSDM: Gasdermin
GSDMD: Gasdermin D
GSDME: Gasdermin E
HAMP: Homeostasis-altering molecular process
HI-FBS: Heat-inactivated fetal bovine serum
HMGB1: High mobility group box 1
iBMDM: Immortalized bone marrow-derived macrophage
IKK: Inhibitor of nuclear factor-κB (IκB) kinase
IMDM: Iscove’s Modified Dulbecco’s Medium
KO: Knockout
LDH: Lactate dehydrogenase
LUBAC: Linear ubiquitin chain assembly complex
MageCK: Model-based analysis of genome-wide CRISPR-Cas9 knockout
MAPK: Mitogen-activated protein kinase
MK-2: Mitogen-activated protein kinase-activated protein kinase 2
MLKL: Mixed lineage kinase domain-like pseudokinase
mtROS: Mitochondrial reactive oxygen species
NF-κB: Nuclear factor kappa B
NLRP3: Nucleotide oligomerization domain, leucine rich repeat and pyrin domain-containing protein 3
NP-40: Nonidet P-40
NT: Non-targeting
PAMP: Pathogen-associated molecular pattern
pMLKL: Phosphorylated mixed lineage kinase domain-like pseudokinase
PP1γ: Protein phosphatase 1 gamma
PP6: Protein phosphatase 6
PPP1R3G: Protein phosphatase 1 regulatory subunit 3G
PPP6C: Protein phosphatase 6 catalytic subunit
PPP6R1: Protein phosphatase 6 regulatory subunit 1
PPP6R2: Protein phosphatase 6 regulatory subunit 2
PPP6R3: Protein phosphatase 6 regulatory subunit 3
pRIPK1: Phosphorylated receptor-interacting serine/threonine protein kinase 1
PRR: Pattern recognition receptor
PVDF: Polyvinylidene fluoride
RET: Reverse electron transport
RIG-I: Retinoic acid-inducible gene I
RIPA: Radioimmunoprecipitation assay
RIPK1: Receptor-interacting serine/threonine protein kinase 1
SDS-PAGE: Sodium dodecyl sulfate polyacrylamide gel electrophoresis
siRNA: Short interfering RNA
STING: Stimulator of interferon genes
TAK1: Transforming growth factor-β–activated kinase 1
TAK1i: Transforming growth factor-β–activated kinase 1 inhibitor
TBK1: TANK-binding kinase 1
TGF-β: Transforming growth factor-β
tMLKL: Total mixed lineage kinase domain-like pseudokinase
TNFR1: Tumor necrosis factor receptor 1
TNFRSF1A: Tumor necrosis factor receptor superfamily member 1A
tRIPK1: Total receptor-interacting serine/threonine protein kinase 1
YopJ: *Yersinia* outer protein J
