# Supplementary figures and images for: The protein phosphatase PP6 promotes RIPK1-dependent PANoptosis
Source: BMC Biol. 2024 May 29;22:122. doi: 10.1186/s12915-024-01901-5 (PMC11134900; doi:10.1186/s12915-024-01901-5)

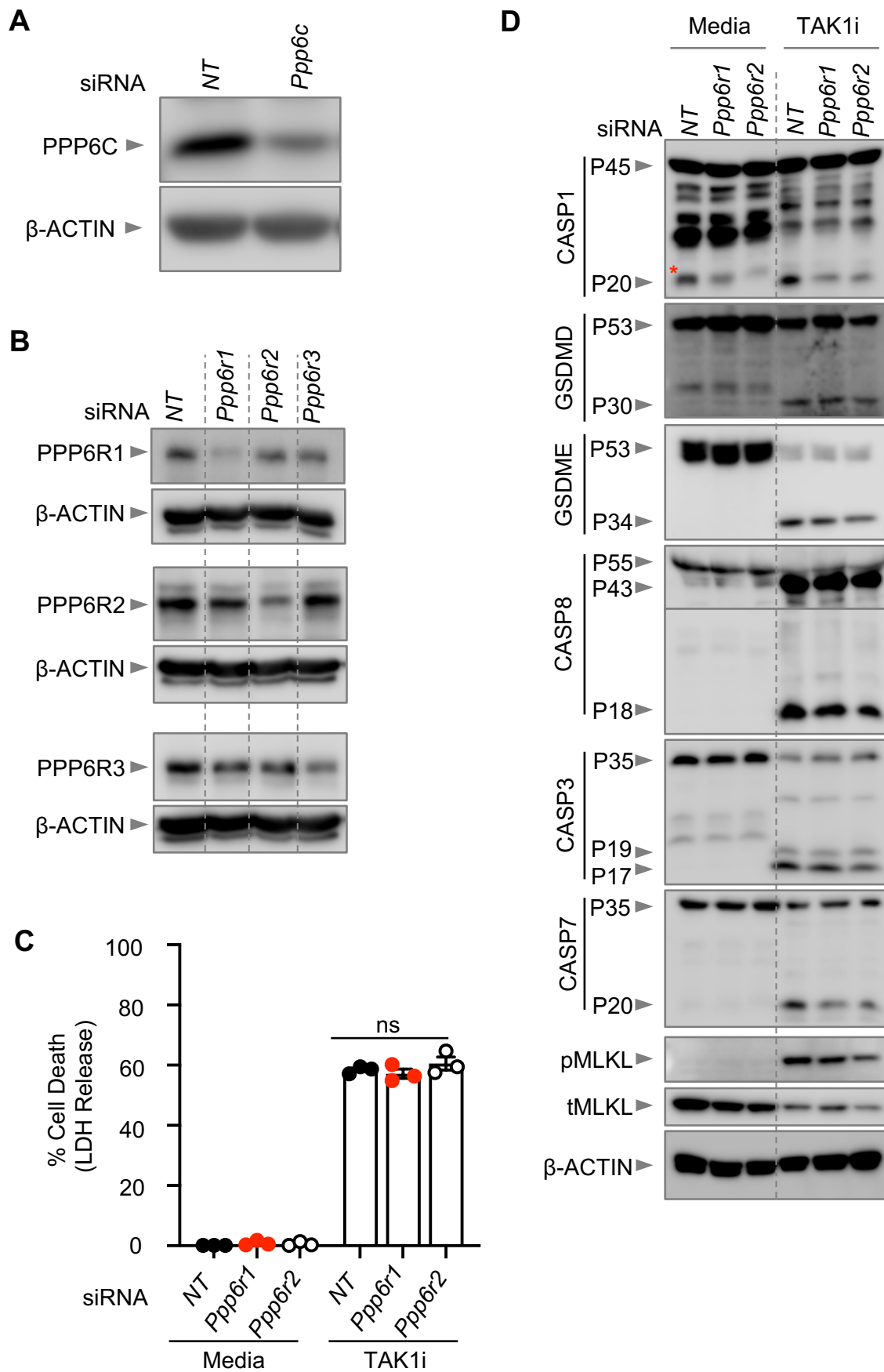

**Fig. S1**

Supplement: Supplementary file 1 — Additional file 1: Figure S1. Individual PP6 complex regulatory subunits do not impact TAK1 inhibition-induced PANoptosis. (A–B) Immunoblots for A) PPP6C and B) PPP6R1, PPP6R2, and PPP6R3 using cell lysates from bone marrow-derived macrophages (BMDMs) following the indicated siRNA knockdowns. Blots were re-probed for β-ACTIN to serve as the internal control. C) Percent cell death as measured by lactate dehydrogenase (LDH) release in BMDMs treated with siRNA against Ppp6r1 or Ppp6r2 in response to TAK1 inhibitor (TAK1i) treatment for 8 h. Statistical analysis was performed using the two-way ANOVA. ns, not significant. D) Immunoblots for pro- (P45) and cleaved caspase-1 (P20; CASP1); pro- (P53) and activated (P30) gasdermin D (GSDMD); pro- (P53) and activated (P34) gasdermin E (GSDME); pro- (P55) and cleaved caspase-8 (P43/18; CASP8); pro- (P35) and cleaved caspase-3 (P19/17; CASP3); pro- (P35) and cleaved caspase-7 (P20; CASP7); and phospho-MLKL (pMLKL) and total MLKL (tMLKL) using cell lysates from BMDMs treated with TAK1i for 8 h. An asterisk indicates a non-specific band near the CASP1 P20 fragment. Blots were re-probed for β-ACTIN to serve as the internal loading control. The data are representative of three independent experiments. NT: non-targeting siRNA. [file 12915_2024_1901_MOESM1_ESM.pdf]
