# Supplementary material for: The protein phosphatase PP6 promotes RIPK1-dependent PANoptosis
Source: BMC Biol. 2024 May 29;22:122. doi: 10.1186/s12915-024-01901-5 (PMC11134900; doi:10.1186/s12915-024-01901-5)

Uncropped blots corresponding to Fig. 2

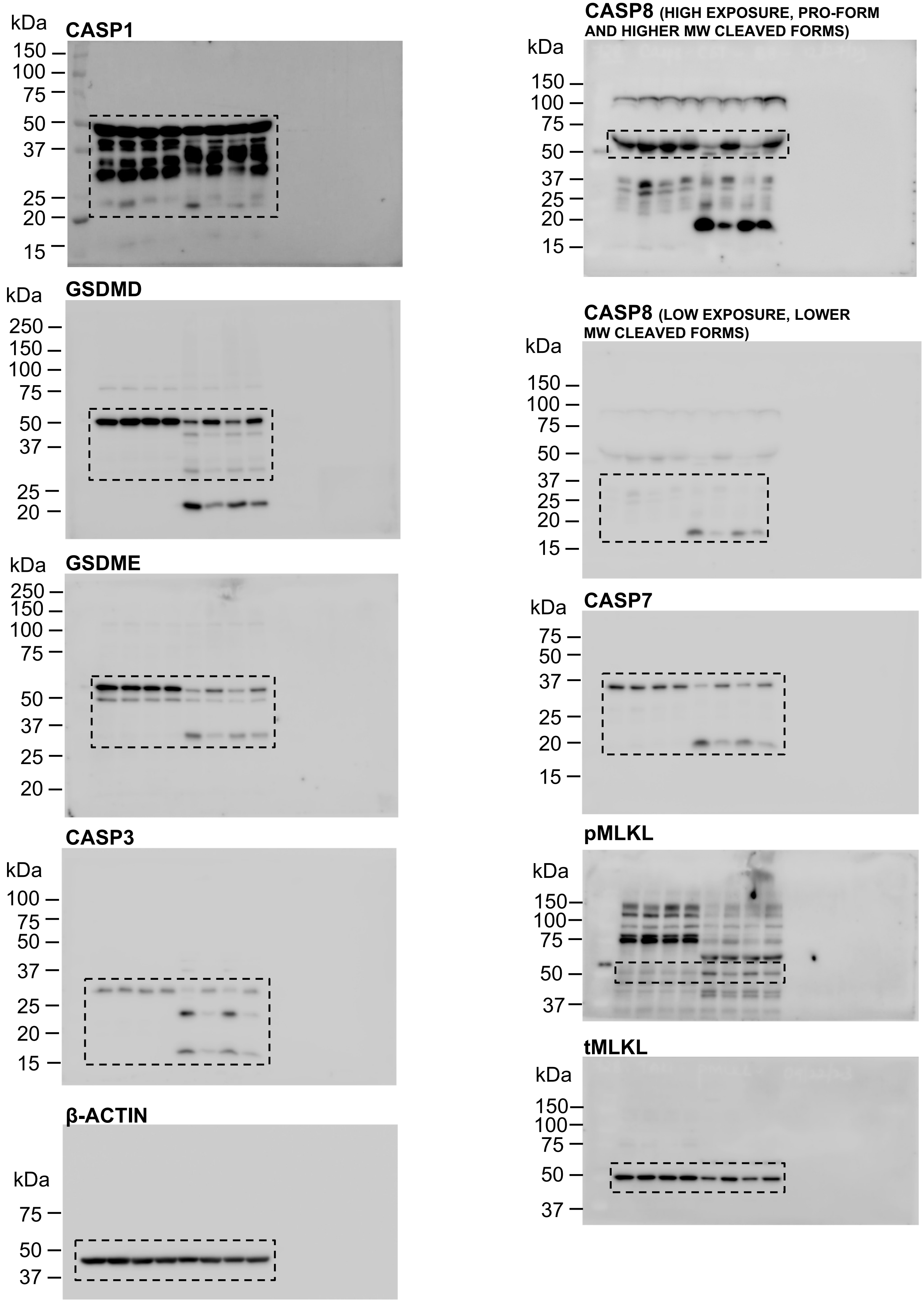

Uncropped blots corresponding to Fig. 3A

S166 pRIPK1

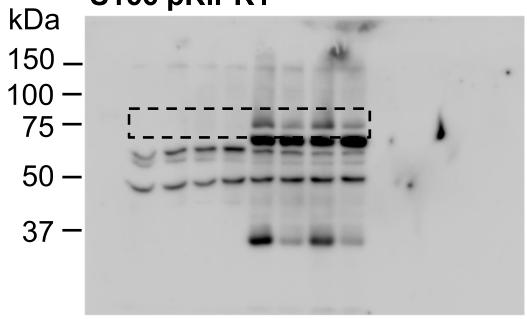

tRIPK1

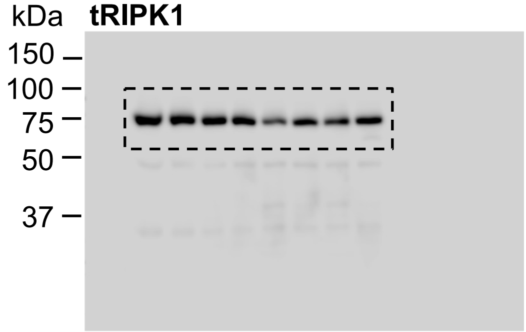

S321 pRIPK1

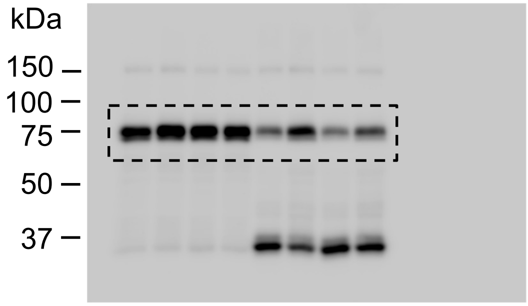

tRIPK1

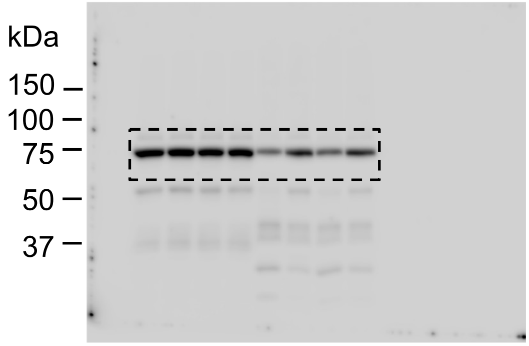

β-ACTIN

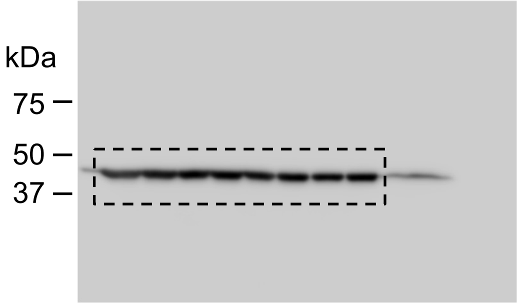

HMGB1

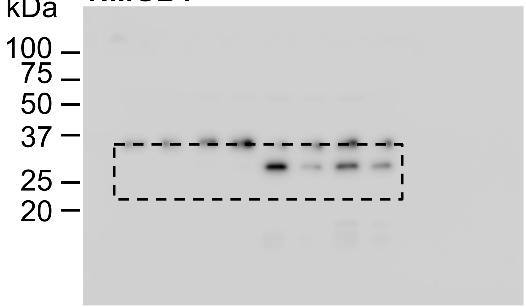

LDH

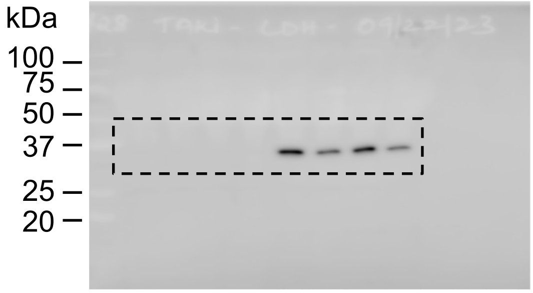

Uncropped blots corresponding to Fig. S1A and 1B

Fig. S1A

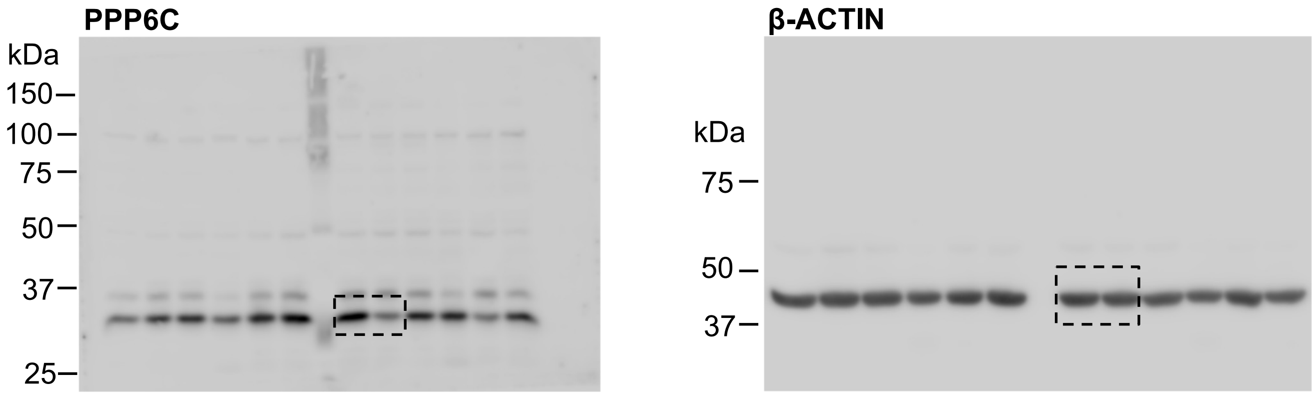

Fig. S1B

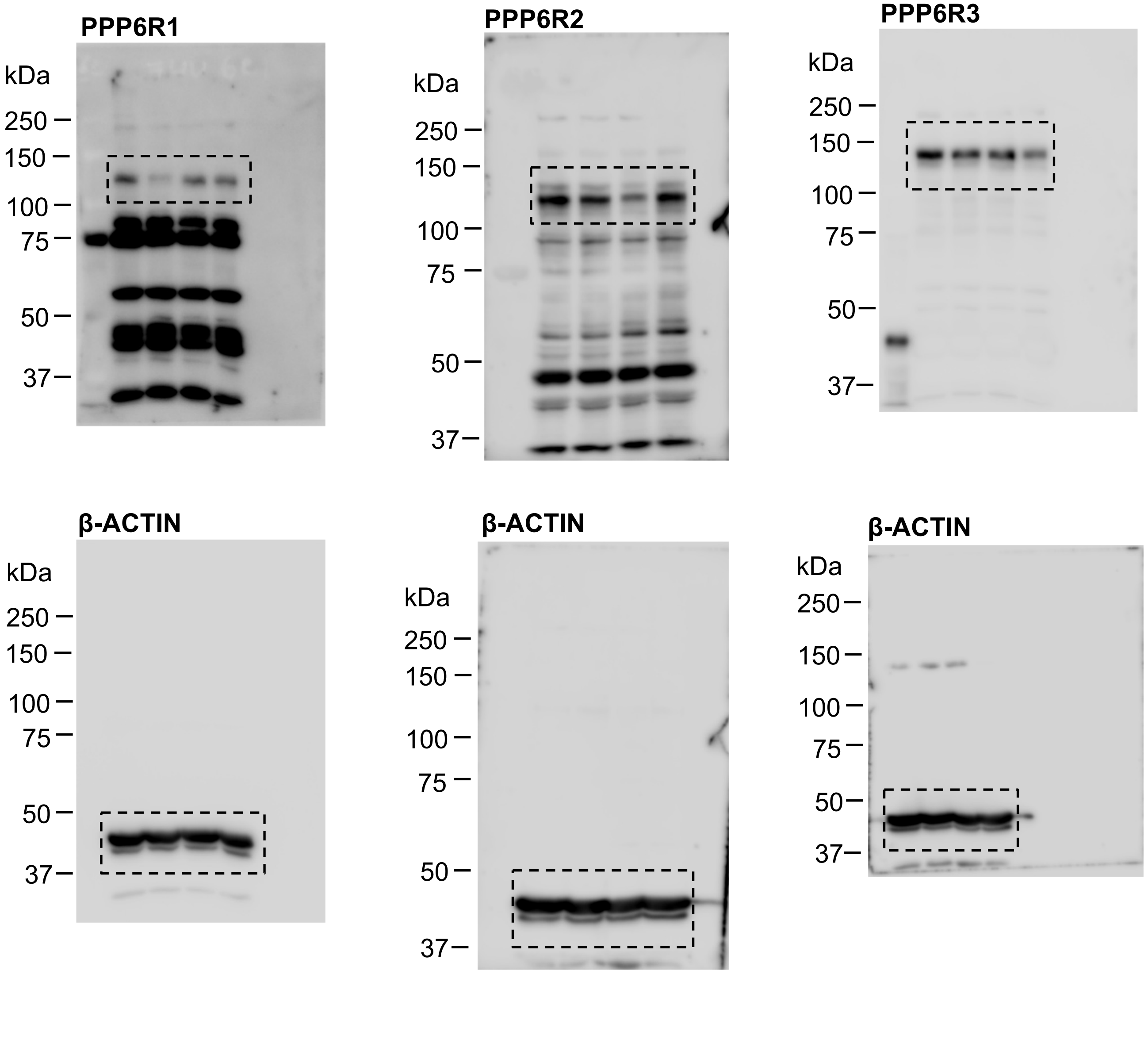

Uncropped blots corresponding to Fig. S1D

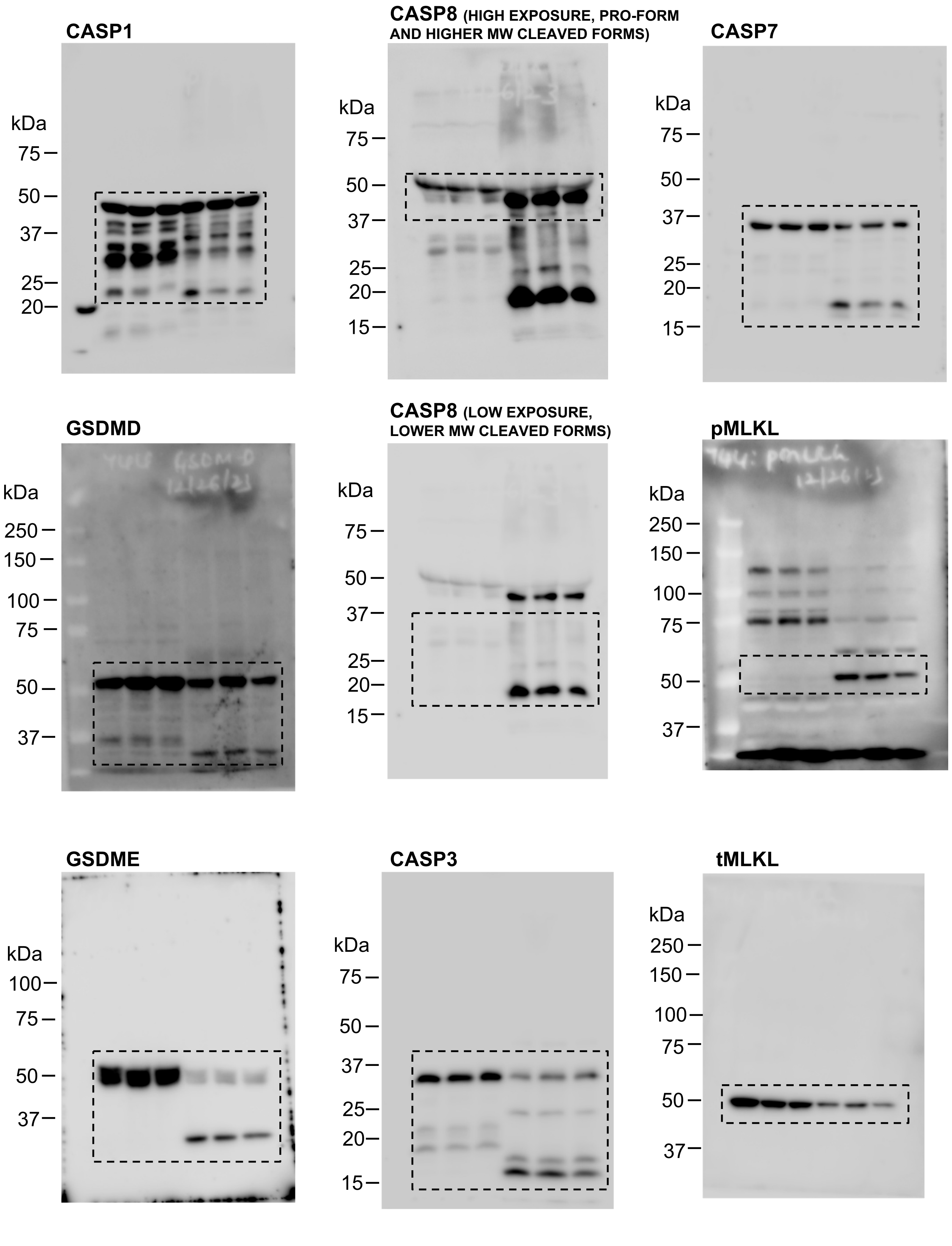

Uncropped blots corresponding to Fig. S1D

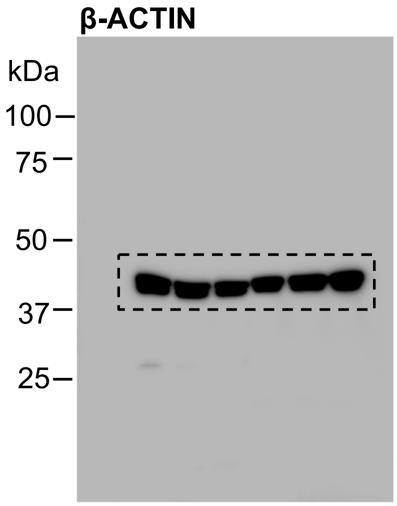

Supplement: Supplementary file 2 — Additional file 2: Uncropped blots. The uncropped, raw blots corresponding with Figs. 2, 3A, S1A, S1B, and S1D are shown with molecular weight indicators. [file 12915_2024_1901_MOESM2_ESM.pdf]
